# Synergistic depletion of gut microbial consortia, but not individual antibiotics, reduces amyloidosis in APPPS1-21 Alzheimer’s transgenic mice

**DOI:** 10.1038/s41598-020-64797-5

**Published:** 2020-05-18

**Authors:** Hemraj B. Dodiya, Mary Frith, Ashley Sidebottom, Yajun Cao, Jason Koval, Eugene Chang, Sangram S. Sisodia

**Affiliations:** 1 Department of Neurobiology, The University of Chicago, Chicago, IL 60637 USA; 2 Department of Medicine, The University of Chicago, Chicago, IL 60637 USA

**Keywords:** Alzheimer's disease, Microbiota, Experimental models of disease

## Abstract

In preceding efforts, we demonstrated that antibiotic (ABX) cocktail-mediated perturbations of the gut microbiome in two independent transgenic lines, termed *APP*_*SWE*_/*PS1*_*ΔE9*_ and APPPS1-21, leads to a reduction in Aβ deposition in male mice. To determine whether these observed reductions of cerebral Aβ amyloidosis are specific to any individual antibiotic or require the synergistic effects of several antibiotics, we treated male APPPS1-21 transgenic mice with either individual ABX or an ABX cocktail and assessed amyloid deposition. Specifically, mice were subject to oral gavage with high dose kanamycin, gentamicin, colistin, metronidazole, vancomycin, individually or in a combination (ABX cocktail) from postnatal days (PND) 14 to 21, followed by *ad libitum*, low-dose individual ABX or ABX cocktail in the drinking water until the time of sacrifice. A control group was subject to gavage with water from PND 14 to 21 and received drinking water till the time of sacrifice. At the time of sacrifice, all groups showed distinct cecal microbiota profiles with the highest differences between control and ABX cocktail-treated animals. Surprisingly, only the ABX cocktail significantly reduced brain Aβ amyloidosis compared to vehicle-treated animals. In parallel studies, and to assess the potential exposure of ABX to the brain, we quantified the levels of each ABX in the brain by liquid chromatography-mass spectrometry (LC-MS) at PND 22 or at 7 weeks of age. With the exception of metronidazole (which was observed at less than 3% relative to the spiked control brains), we were unable to detect the other individual ABX in brain homogenates. Our findings suggest that synergistic alterations of gut microbial consortia, rather than individual antimicrobial agents, underlie the observed reductions in brain amyloidosis.

## Introduction

Extracellular amyloid plaques composed of Aβ peptides and intraneuronal inclusions of tau in neurofibrillary tangles are the pathological hallmarks of Alzheimer’s disease^1,2^. In addition to these proteinaceous aggregates, production of proinflammatory cytokines^3–5^ and the presence of plaque-localized microglia^6–8^ and astrocytes^9^ are believed to play a major role in Alzheimer’s-related neurodegeneration. Although inflammatory responses are intended to be protective, chronic excessive inflammatory responses can cause, or contribute to, both spreading of pathology and tissue damage^10^.

We have shown that ABX-perturbed gut microbiome in two different transgenic mouse models of Aβ amyloidosis leads to a sex-specific reduction of brain amyloidosis in male mice^11,12^. Furthermore, transplantation of fecal content from transgenic mice into ABX-treated male APPPS1-21 mice restores amyloid deposition thus establishing a causal link between the gut microbiome and Aβ amyloidosis^12^. While the exact mechanism(s) by which the gut microbiome influences Aβ amyloidosis have not been clarified, we have provided support for a role of microglial activation^11–13^, but may also involve endocrine signaling^14^ or pathological seeding^15^ amongst others.

In order to explore the microbiome-brain axis in Alzheimer’s disease, the field has relied on experimental approaches including re-derivation to generate germ-free (GF) AD mice^16^, fecal microbiota transfer (FMT) between animals^17,18^, treating AD mice with one or more defined microbial strains^19,20^ or antibiotics^11,12,21^. Of these, the simplest approach to perturb the gut microbiome is to treat animals with ABX. Indeed, this approach, that we term “pseudo-germ-free mice” has been widely used to assess the impact of alterations in the microbiome in the fields of immunology and metabolism^22–26^. Despite the gain in popularity of pseudo-germ-free mice models to explore the role of gut microbiome in different conditions, there are limitations. While it is assumed that the effects of antibiotic treatment on disease manifestations are mediated by the gut microbiome, recent evidence suggests that this causal relationship is not always the case. For example, ABX can directly affect peripheral immune cells to ameliorate intestinal inflammation in both specific pathogen free (SPF) and GF mouse models of dendritic cell-specific TRAF6 knock-out mice^27^. Similarly, ABX have been shown to directly suppress mitochondrial and ribosomal functions in GF mice treated with ABX^28^. Thus, we felt it was critical to evaluate our pseudo-germ-free mouse models of Aβ amyloidosis to allay these concerns.

To validate the use of our pseudo-germ-free mouse models of Aβ amyloidosis, we sought to address the following: (1) How do alterations in the gut microbiome in animals treated with individual ABX affect brain amyloidosis? and (2) do ABX affect brain amyloidosis by directly entering the brain and altering Aβ metabolism in a manner independent of alterations in the gut microbiota? To address the first issue, we investigated the impact of treating APPPS1-21 animals with individual antibiotics or the ABX cocktail in male mice only. The rationale for the use of male mice was based on our previously published results^11,12^ in which we demonstrated that in two independent transgenic mouse lines (APP_SWE/_PS1_ΔE9_ and APPPS1-21(APP_SWE_/PS1_L166P_ mice), only male mice showed reduced Aβ amyloidosis following administration of the ABX cocktail. We observed specific microbiota alterations in cecal contents of animals treated with individual ABX, and as expected, the differences in microbiota profiles were the highest between ABX cocktail-treated male mice compared with vehicle or individual ABX-treated mice. Importantly, microbiota changes were associated with significantly reduced amyloidosis in mice treated with ABX cocktail, but cohorts of animals treated with each of the individual antibiotics had no discernable effect on brain amyloidosis compared with animals treated with vehicle. To address the second issue, we employed LC-MS to evaluate the presence of individual ABX in brain lysates of animals treated with individual ABX. These analyses revealed that with the exception of metronidazole, that accumulated a very minimal levels at both PND 22 and 7 weeks of age, none of the other ABX were detectable in brain lysates at these time points. Importantly, the minimal brain exposure of metronidazole had no impact on levels of amyloid deposition. Collectively, these findings lead us to suggest that by mechanism(s) presently uncertain, alterations in microbial composition, but not brain exposure of each antibiotic are responsible for the observed reductions in Aβ amyloidosis in our mouse model.

## Results

### Long-term individual antibiotic treatment resulted in specific changes in cecal size and cecal microbiome profile

**Figure 1:**
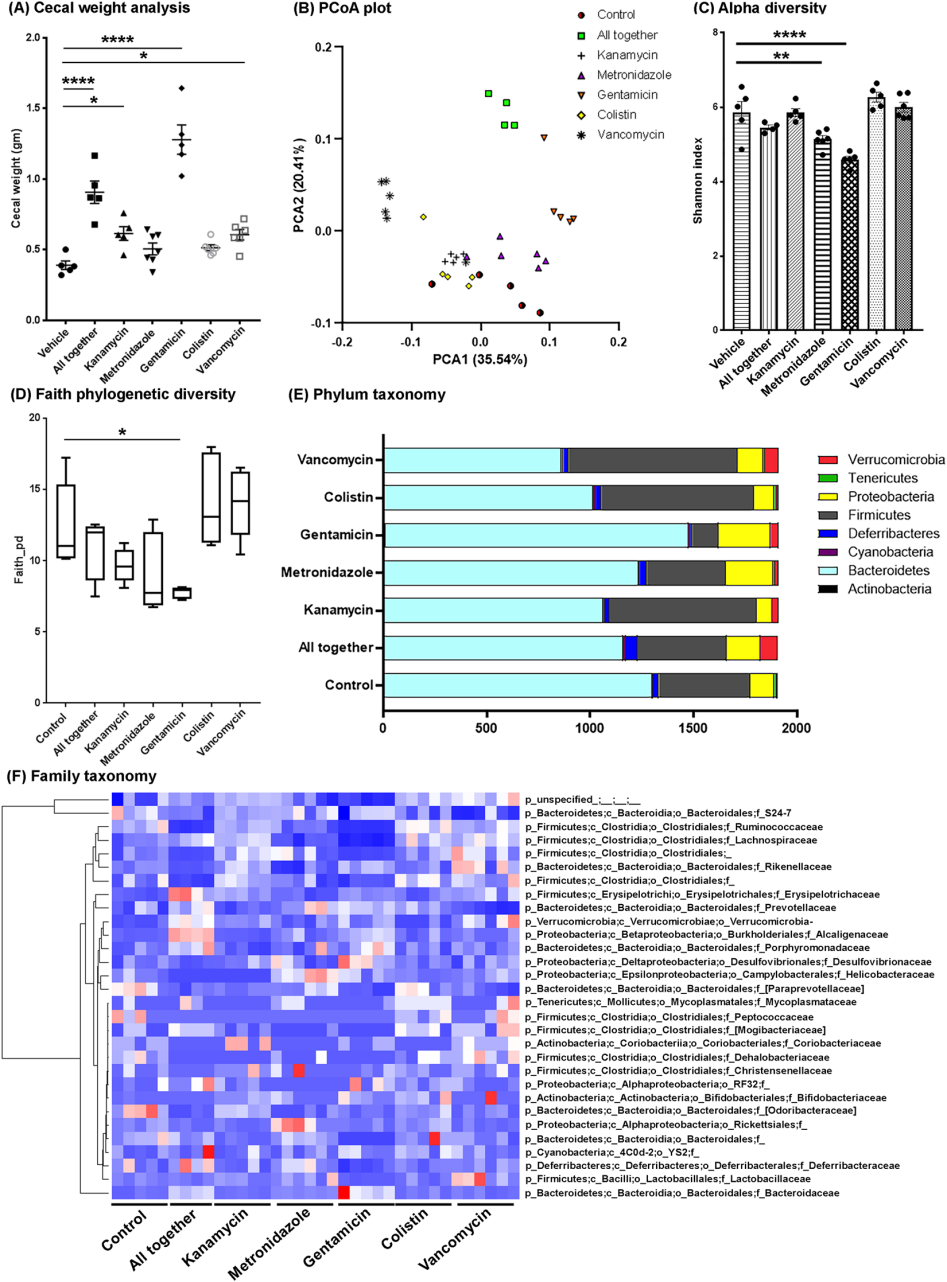
Cecal weights and microbiome profile is altered after individual or combinatorial ABX (ABX-cocktail) at 9 weeks of age. (**A**) Cecal weights from vehicle-, ABX-cocktail or individual ABX-treated male mice. One-way ANOVA showed significant differences among groups (F(6,32) = 29.2, *P* < 0.0001). Post-hoc analysis showed ABX cocktail (“All together”) (*P* = 0.0001), kanamycin (*P* = 0.045), gentamicin (*P* = 0.0001) and vancomycin (*P* = 0.044) treatment resulted in significantly larger cecal weight compared with control. N = 5 control (vehicle), n = 5 kanamycin, n = 7 metronidazole, n = 5 gentamicin, n = 6 colistin, n = 6 vancomycin, n = 5 abx cocktail treated male mice. (**B**) PCoA plot of abundance-weighed Unifrac distances, a metric of beta diversity. The two components (PCA1, PCA2) explained 35.54% and 20.41% of the variance, respectively. ABX cocktail showed a clear separation compared with the control mice. Gentamicin- and vancomycin- treated-mice also showed a clear separation compared with control. (**C**,**D**) Alpha-diversity differed significantly across groups (One-way ANOVA: F(6,29) = 15.45, *P* < 0.0001) and was measured using the Shannon diversity index, which accounts for both community evenness and richness. Specifically, metronidazole (*P* < 0.0001) and gentamicin (*P* < 0.0001)-treated groups showed significantly reduced alpha diversity compared to control. (**D**) Phylogenetic richness also differed significantly across groups (One-way ANOVA: F(6,29) = 5.814, *P* = 0.0005) and was measured using the Faith *P*hylogenetic Diversity Index. Post hoc tests indicated that the gentamicin-treated group had significantly lower phylogenetic richness compared to control (*P* = 0.0215). (**E**,**F**) Microbiota profile representing the (**E**) mean observed abundances at the phylum level and (**F**) relative abundances at the family level (taxa comprising <1% abundance in all samples are not shown) in vehicle-, ABX-cocktail or individual ABX-treated male mice. N = 5 control (vehicle), n = 5 kanamycin, n = 6 metronidazole, n = 5 gentamicin, n = 5 colistin, n = 6 vancomycin, n = 4 ABX cocktail treated male mice were used for microbiota analyses. Data are mean ± SEM. *P < 0.05, **P < 0.01, ****P < 0.0001.

Male APPPS1-21 mice were subject to oral gavage with high dose kanamycin, gentamicin, colistin, metronidazole, vancomycin, ABX-cocktail, or vehicle (water) from PND 14 to 21, followed by *ad libitum*, low-dose (1/50^th^ gavage dose) individual ABX or ABX-cocktail in the drinking water until the time of sacrifice. To determine whether individual ABX treatment leads to cecal enlargement as was seen with ABX-cocktail treatment^12^, ceca were weighed at the time of sacrifice and revealed that ABX-cocktail treated mice were significantly larger than those of vehicle-treated animals (*P* < 0.05; Fig. 1A). However, we also observed significantly enlarged ceca in kanamycin, gentamicin and vancomycin-treated mice groups compared with mice treated with vehicle (*P* < 0.05).

The cecal microbiota of each animal was profiled using Illumina MiSeq. 16S rRNA amplicons, revealing significant differences between all groups. From beta diversity analyses, pairwise PERMANOVA of abundance-weighted and unweighted Unifrac distances revealed significant differences between all pairs of groups, with the exception of colistin and water (weighted, *P* = 0.094; unweighted *P* = 0.185), metronidazole and water (unweighted only, *P* = 0.065), and colistin and vancomycin (unweighted only, *P* = 0.184). Weighted Unifrac results are shown as a principle coordinate analysis (PCoA) plot in Fig. 1B and demonstrate clear separation of clusters between vehicle compared with ABX cocktail-treated male mice. Additionally, individual ABX-treated groups vancomycin and gentamicin showed farthest separation of clusters compared with the vehicle-treated group. The clusters for the kanamycin- and colistin-treated groups showed minimal separation suggesting similar microbiota profiles. Shannon alpha diversity indices were significantly lower for metronidazole- and gentamicin-treated groups compared with the vehicle-treated group, although only the gentamicin-treated group reached statistical significance when compared with control using Faith’s phylogenetic diversity index (Fig. 1C,D). Taxonomy abundances at the phylum and family level showed very distinct profiles between groups (Fig. 1E,F). Taxonomic abundances in ABX cocktail-treated mice showed changes in *s_Uniformis*, Lachnospiraceae (family), Parabacteroides (genus), Ruminococcus (genus) (Table 1, Fig. 1F), that are similar to those we observed previously^12^. Furthermore, individual ABX treatments exhibited differences compared with the ABX cocktail with respect to their effects on taxonomic abundances relative to vehicle treatment as assessed by Analysis of Composition of Microbiomes (ANCOM). Specifically, kanamycin resulted in higher Clostridiales at the exact sequence variant (ESV) level, and lower Helicobacteraceae family members after sequences were clustered up to the species level (L7 clustered taxa). Metronidazole resulted in reduced abundance of Ruminococcus at ESV level, and Lactobacillus, Odoribacter and Desulfovibrio C20 taxa at L7 clustered taxa level. Gentamicin resulted in higher *Bacteroides uniformis*, Parabacteroides, Lachnospiraceae, *Blautia producta* at ESV level with higher *Eubacteria dolichum* and lower Oscillospira, Coprococcus, and Desulfovibrio C20 at L7 clustered taxa. Vancomycin resulted in higher *Ruminococcus gnavus* and lower Oscillospira at ESV level with no changes at L7 clustered taxa. Colistin did not result in any significant differences in both ESV and L7 clustered taxa (Table 1).

**Table 1:** QIIME 2 ANCOM analysis of microbial taxa in each ABX-treated group versus Vehicle-treated controls.

	Higher than Vehicle	Lower than Vehicle
**Abx Cocktail vs Vehicle**		
ESVs	p_Bacteroidetes; c_Bacteroidia; o_Bacteroidales; f_Bacteroidaceae; g_Bacteroides; s_uniformis	
	p_Firmicutes; c_Clostridia; o_Clostridiales; f_Lachnospiraceae	
L7 Clustered Taxa	p_Bacteroidetes;c_Bacteroidia;o_Bacteroidales;f_Bacteroidaceae;g_Bacteroides;s_uniformis	p_Firmicutes;c_Clostridia;o_Clostridiales;f_Ruminococcaceae;g_Ruminococcus;s_
	p_Bacteroidetes;c_Bacteroidia;o_Bacteroidales;f_Porphyromonadaceae;g_Parabacteroides;_	p_Proteobacteria;c_Deltaproteobacteria;o_Desulfovibrionales;f_Desulfovibrionaceae;g_Desulfovibrio;s_C21_c20
	p_Proteobacteria;c_Deltaproteobacteria;o_Desulfovibrionales;f_Desulfovibrionaceae;g_Desulfovibrio;s_	p_Firmicutes;c_Clostridia;o_Clostridiales;f_Ruminococcaceae;g_;s_
		p_Bacteroidetes;c_Bacteroidia;o_Bacteroidales;f_Porphyromonadaceae;g_Parabacteroides;s_
**Kanamycin vs Vehicle**		
ESVs	p_Firmicutes; c_Clostridia; o_Clostridiales	
L7 Clustered Taxa		p_Proteobacteria;c_Epsilonproteobacteria;o_Campylobacterales;f_Helicobacteraceae;_;_
**Metronidazole vs Vehicle**		
ESVs		p_Firmicutes; c_Clostridia; o_Clostridiales; f_Ruminococcaceae; g_Ruminococcus; s_
L7 Clustered Taxa		p_Firmicutes;c_Clostridia;o_Clostridiales;f_Ruminococcaceae;g_;s_
		p_Firmicutes;c_Bacilli;o_Lactobacillales;f_Lactobacillaceae;g_Lactobacillus;_
		p_Bacteroidetes;c_Bacteroidia;o_Bacteroidales;f_[Odoribacteraceae];g_Odoribacter;s_
		p_Proteobacteria;c_Deltaproteobacteria;o_Desulfovibrionales;f_Desulfovibrionaceae;g_Desulfovibrio;s_C21_c20
**Gentamicin vs Vehicle**		
ESVs	p_Bacteroidetes; c_Bacteroidia; o_Bacteroidales; f_Bacteroidaceae; g_Bacteroides; s_uniformis	
	p_Bacteroidetes; c_Bacteroidia; o_Bacteroidales; f_Porphyromonadaceae; g_Parabacteroides	
	p_Firmicutes; c_Clostridia; o_Clostridiales; f_Lachnospiraceae; g_Blautia; s_producta	
L7 Clustered Taxa	p_Bacteroidetes;c_Bacteroidia;o_Bacteroidales;f_Bacteroidaceae;g_Bacteroides;s_uniformis	p_Firmicutes;c_Clostridia;o_Clostridiales;f_Ruminococcaceae;g_Oscillospira;s_
	p_Bacteroidetes;c_Bacteroidia;o_Bacteroidales;f_Porphyromonadaceae;g_Parabacteroides;_	p_Firmicutes;c_Clostridia;o_Clostridiales;f_Lachnospiraceae;g_Coprococcus;s_
	p_Firmicutes;c_Clostridia;o_Clostridiales;f_Lachnospiraceae;g_Blautia;s_producta	p_Firmicutes;c_Clostridia;o_Clostridiales;f_Lachnospiraceae;_;_
	p_Firmicutes;c_Erysipelotrichi;o_Erysipelotrichales;f_Erysipelotrichaceae;g_[Eubacterium];s_dolichum	p_Firmicutes;c_Clostridia;o_Clostridiales;f_Lachnospiraceae;g_;s_
		p_Proteobacteria;c_Deltaproteobacteria;o_Desulfovibrionales;f_Desulfovibrionaceae;g_Desulfovibrio;s_C21_c20
		p_Bacteroidetes;c_Bacteroidia;o_Bacteroidales;f_;g_;s_
**Colistin vs Vehicle**		
ESVs & L7 Clustered Taxa - *No significant differences*		
Vancomycin vs Vehicle		
ESVs	p_Firmicutes; c_Clostridia; o_Clostridiales; f_Lachnospiraceae; g_[Ruminococcus]; s_gnavus	p_Firmicutes; c_Clostridia; o_Clostridiales; f_Ruminococcaceae; g_Oscillospira; s_
L7 Clustered Taxa	*No significant differences*	

### No impact of individual ABX on cerebral amyloidosis

**Figure 2:**
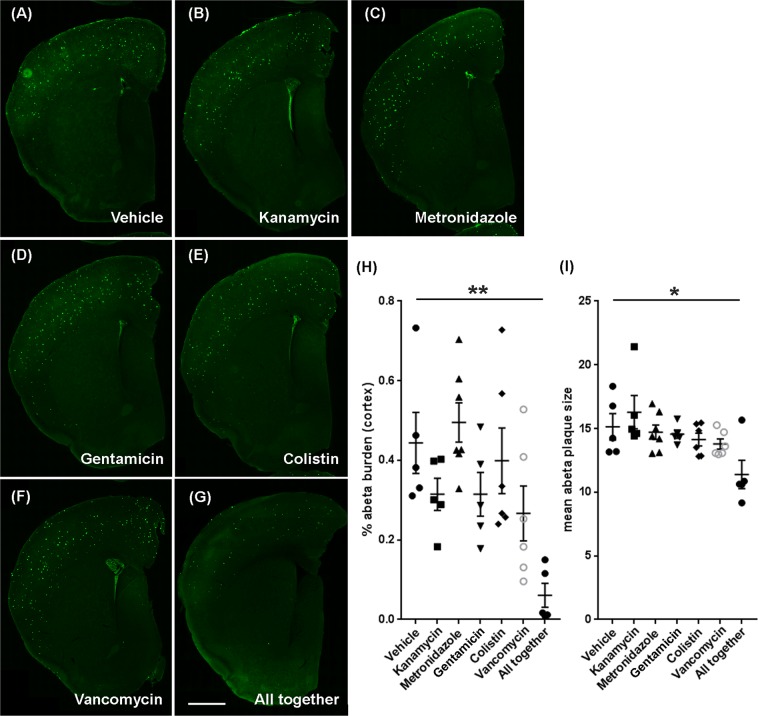
Reduced Aβ pathology was specific to the ABX-cocktail (all together)-treated male mice at 9 weeks of age. Representative images of Aβ plaque burden in the Vehicle (**A**), kanamycin (**B**), metronidazole (**C**), gentamicin (**D**), colistin (**E**), vancomycin (**F**), and ABX-cocktail (all together) (**G**) treated male mice using 3D6 antibody. (**H**) Aβ burden (*P* = 0.0012) and (**I**) Aβ plaque size (*P* = 0.0146) was significantly lower in ABX-cocktail (all together) treated male mice (One-way ANOVA: Aβ burden: F(6, 32) = 5.276, *P* = 0.0007; Aβ plaque size: F(6, 32) = 3.394, *P* = 0.0105), while individual ABX-treated mice showed no significant differences (*P* > 0.05). Scale bar in panel g represents 1000 µm and applies to all panels a-g. N = 5 control (vehicle), n = 5 kanamycin, n = 7 metronidazole, n = 5 gentamicin, n = 6 colistin, n = 6 vancomycin, n = 5 ABX cocktail- treated male mice. Data are mean ± SEM. *P < 0.05, **P < 0.01.

Having established distinct perturbations in cecal microbial compositions between animals treated with individual ABX or ABX cocktail, we then investigated the impact on cerebral Aβ amyloidosis, Mice were sacrificed at 9 weeks of age, and Aβ-specific 3D6 antibody was used for IHC as previously described^12^. As we reported earlier^12^, ABX cocktail-treated male APPPS1-21 mice showed significantly reduced amyloidosis (Fig. 2A,G,H) and smaller amyloid plaque size (Fig. 2A,G,I) in the cerebral cortex compared with vehicle-treated animals (*P* < 0.05). However, we did not observe any significant differences in cerebral cortex amyloidosis between individual ABX-treated mice groups compared with vehicle-treated mice (*P* > 0.05) (Fig. 2A–I).

### Accumulation of individual antibiotics in brain homogenates of treated mice

**Figure 3:**
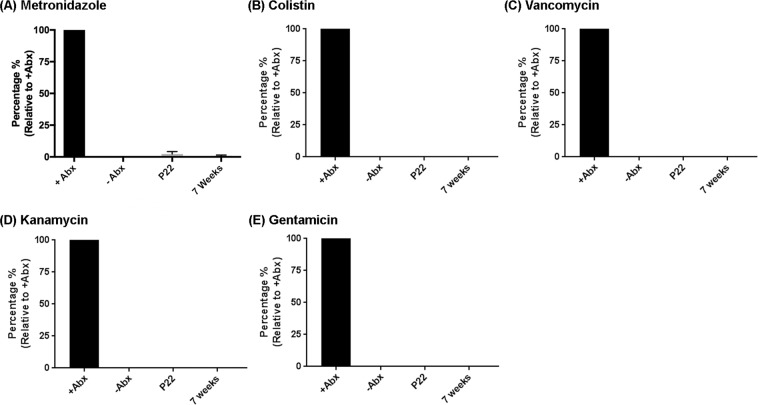
Metronidazole was present at very low levels in the brain parenchyma at both post-natal day (PND) 22 and 7 weeks of age. LC-MS was performed to investigate the ABX level in the brain. (**A**) Metronidazole in low level (less than 3%) was detected in brain homogenate extract while other ABX (colistin (**B**), vancomycin (**C**), kanamycin (**D**), gentamicin (**E**)) were below the detectable range (LOD) in the brain homogenate extracts. N = 6/group, Data are mean ± SEM.

To examine the accumulation of individual antibiotics in the brains of treated male mice, 5 animals/group were sacrificed at PND 22 or at 7 weeks of age. Brains were harvested and processed using tissue homogenizer using Milli-Q water to collect the supernatant fraction. The supernatant fractions were then loaded on to an activated strata column to extract ABX according to the manufacturer-recommended protocol. These extracts were processed through an LC-MS instrument to measure the levels of individual ABX in the brain. Brains were spiked with individual ABX at specific doses and went through the same extraction protocol. Relative concentrations were extrapolated comparing the spiked samples for the documentation. Metronidazole showed the presence in the brain extract at both post-natal day 22 and 9 weeks of age, albeit at less than 3% relative to the spiked sample (Fig. 3A) while the other antibiotics were not detectable in the brain (Fig. 3B–E).

## Discussion

Several studies have reported changes in the gut microbiota in AD subjects and cognitively impaired adults^29,30^ that are correlated with gut microbiota dysbiosis. Similarly, altered gut microbiota are evident in animal models of AD^31–33^. Despite these strong correlations, it was not clear if the observed changes in gut microbiota are a cause or consequence of Alzheimer’s disease. Manipulation of the gut microbiome either by treating animals with ABX for their entire lifetime or only postnatally was associated with reduced amyloid plaque pathology in male mice only^11–13^. Most importantly, we recently established causality between gut microbiome and Alzheimer’s plaque pathology using fecal transplantation (FMT). Specifically, FMT from transgenic mice to ABX-treated APPPS1-21 mice partially restored ABX-perturbed gut microbiota and brain amyloidosis^12^. Besides our group, others have employed different broad-spectrum antibiotic agents in a different transgenic line of Aβ amyloidosis and have reported effects on peripheral immune cell infiltration and microglial activation^21^.

In the past, several studies evaluated minocycline, a tetracycline-derived antibiotic (molecular mass ~450 Da) using preclinical AD models. These studies reported that minocycline was able to cross the blood-brain barrier (BBB), inhibit proinflammatory microglia, Aβ fibrillization, tau phosphorylation and neuronal cell death^34–37^. Similar to the effect of minocycline, the most important concern in our pseudo-germ-free model was to differentiate the effect of antibiotics in the brain irrespective of the influence of alterations of the microbiome. To address this concern, we provided individual antibiotics to male APPPS1-21 mice and evaluated cortical Aβ plaques. We found that none of the antibiotics that were used in our ABX-cocktail, when provided individually, had any influence on cerebral Aβ amyloidosis. Second, we evaluated the levels of each antibiotic in the cerebral cortex at both PND22 and 7 weeks of age. With the exception of metronidazole that is detectable, albeit at very low levels in the brain, kanamycin, gentamicin, vancomycin and colistin levels were below the limit of detection (LOD) values in the brain at both PND22 and 7 weeks of age, thus confirming their poor permeability through the BBB.

Importantly, individual ABX showed a distinct effect on the gut microbiota profile. At the phylum taxonomy level (Fig. 1E), vancomycin treatment resulted in decreased Bacteroidetes, increased Proteobacteria and increased Verrucomicrobia in APPPS1-21 male mice, observations similar as reported previously in C57BL/6 mice treated with vancomycin (0.5 mg/mL in drinking water for three weeks)^38^. In contrast, gentamicin treatment showed expansion of Bacteroidetes and Proteobacteria at the expense of Firmicutes compared with vehicle or vancomycin treated groups. Metronidazole and colistin treatments resulted in minimal changes in the phylum taxonomy. Most importantly, ABX-cocktail treatment showed the most differences in all phylum taxonomy which was associated with reduced cerebral Aβ amyloidosis in male mice. Future studies employing metagenomic and metabolomic techniques will allow us to investigate the mechanism(s) by which gut microbiota or metabolites contribute to sex-specific amyloid reduction in our APPPS1-21 pseudo-germ-free mouse model.

## Methods

### Animal housing and handling

APPPS1-21 mice in a C57BL6Cj background were obtained from M. Jucker (University of Tubingen, Tubingen, Germany) and were housed in the animal research center facility at the University of Chicago. Mice were housed under standard conditions with *ad libitum* food and water unless otherwise noted. The APPPS1-21 mice co-express the APP_SWE_ and PS1_L166P_ transgenes driven by the neuron-specific Thy1 promoter and exhibit Aβ deposition in the cerebral cortex as early as 6 weeks of age^39^. We have demonstrated that transgene expression is restricted to the brain and that Aβ peptides are present exclusively in this compartment^12^.

All experimental procedures were approved by Institutional Animal Care and Use Committee at the University of Chicago. The experimental methods were carried out in accordance with these guidelines and regulations.

### ABX treatment

Antibiotics were administered to male APPPS1-21 mice as published earlier^12^. In brief, pups were subject to oral gavage with 200 µL of ABX cocktail (4 mg/mL kanamycin (Cat#K4000-5g, Sigma-Aldrich), 0.35 mg/mL gentamicin (Cat#G1914-250mg, Sigma-Aldrich), 8,500 U/mL colistin (Cat#C4461-1g, Sigma-Aldrich), 2.15 mg/mL metronidazole (Cat#M1547-25g, Sigma-Aldrich), and 0.45 mg/mL vancomycin (Cat#V2002-1g, Sigma-Aldrich)) from PND 14 to 21 followed by an *ad libitum* access to freshly prepared 1:50 diluted ABX water until the time of sacrifice at 9 weeks of age. Pups receiving individual ABX were subject to oral gavage with an individual antibiotic followed by an *ad libitum* access to freshly prepared 1:50 diluted individual ABX water until the time of sacrifice. ABX-containing water was changed every week. During the gastric gavage delivery, all pups were transferred to a clean cage to avoid microbial contamination from accumulated fecal pellets in cages. Parents from the same cage as pups receiving ABX treatment were euthanized after weaning the pups and were not used further for breeding. Based on our previous results^12^ showing ABX-mediated reduction in Aβ amyloidosis only in male mice, we only utilized male pups for the current experiments. Specifically, male pups from two/three litters (n = 10) were combined together and were assigned to each treatment groups as mentioned above. We aimed to use minimum of n = 5 mice per group but based on the genotyping results at PND19, we utilized all heterozygous APP_SWE_/PS1_L166P_ male pups. This resulted in n = 5 control (vehicle), n = 5 kanamycin, n = 7 metronidazole, n = 5 gentamicin, n = 6 colistin, n = 6 vancomycin, n = 5 ABX cocktail mice.

### Necropsy and tissue harvesting

Protocols approved by Animal Care and Use committee were followed for necropsy and tissue harvesting as published previously^12^. Mice were brought to a deep anesthesia stage using a mixture of ketamine and xylazine. Blood was collected transcardially using a 25-gauge needle and stored in buffered sodium citrate blood collection tubes (Cat# 366393; BD Vacutainer) on ice. After assessing and clamping descending aorta, mice were perfused by using cold saline (pH 7.4) for 3 min. Brains were then excised and dissected into two hemispheres (left hemisphere was post-fixed with 4% paraformaldehyde and right hemisphere was frozen). Cecum was collected and weighed after careful removal from the small and large intestine. Cecum and large intestine were frozen immediately and stored in −80 °C for future use. Blood was then centrifuged at 2,000 rpm for 10 min at 4 °C by using a Beckman Coulter centrifuge to collect plasma. Plasma was then stored at −80 °C for the future use.

### Cecal microbiota analysis

50–100 mg of cecal content was used to measure microbiota profiles using Illumina MisSeq 16S analysis. Extraction of microbial DNA was performed using Qiagen DNeasy PowerSoil Kit following the manufacturer’s instructions. The cecal content of 5 control- (vehicle), 5 kanamycin-, 6 metronidazole-, 5 gentamicin-, 5 colistin-, 6 vancomycin-, 4 ABX cocktail-treated male mice resulted in successful DNA extraction. Due to unforeseen technical issues, we lost one sample in the metronidazole-, colistin- and ABX cocktail-treated groups. Purified DNA was submitted to Argonne National Laboratories for 16S rRNA amplicon sequencing (Illumina MiSeq). For analysis, raw sequences in Earth Microbiome Project (EMP) format (paired-end reads) were imported into Qiime2^40^, demultiplexed and quality controlled using Dada2^41^. Sequences were aligned using mafft^42^ and a phylogenetic tree was constructed using fasttree^43^. For diversity metrics, sampling depth was rarefied at 1912 sequences per sample to maximize depth while prioritizing equal retention of samples across groups^44^. Alpha diversity metrics (Shannon, Faith^45^) and beta diversity metrics (weighted and unweighted Unifrac distances^46,47^) were calculated. A taxonomy was compiled using the classify-sklearn plugin with the Greengenes 13_8 99% OTUs pre-trained Naïve Bayes classifier^48–50^. To assess differentially abundant taxa at the species level (L7) or among exact sequence variants (ESV) between each treatment and control group, Analysis of Composition of Microbiomes (ANCOM) was performed using the ancom plugin^51^.

### Immunocytochemistry

Histology was performed in a similar fashion as published^12^. After 24 hours of post-fixation with 4% paraformaldehyde, brains were kept in 30% sucrose until the time of sectioning. Using Leica microtome (Cat#SM210R, Leica), the hemi-brains were cut at 40 µm thickness and stored in cryoprotectant solution. A total of 6 level-matched sections at 480 µm intervals were selected from each case. Sections were washed with dilution media for 60 min (10 min wash × 6) and blocked with serum blocking solution for 1 hour. Primary antibody (3D6, 1:10000) was used for overnight incubation followed by washes and secondary antibody (Donkey anti-mouse 488, 1:500, Cat#A-21202, ThermoFisher) incubation for one hour the next day. The sections were then washed with TBS and mounted on glass slides. Mounted slides were then coverslipped using aquamount mounting media (Cat#F4680, ThermoFisher) and dried at the room temperature. Slides were then stored in refrigerator until the time of microscopic analysis.

### Aβ burden analysis

Amyloid quantification was performed as published^12^. Briefly, slides were scanned using a slide scanner at 20x magnification to prepare a 3D Z-stack of each slide. Images of individual sections (n = 6 sections per mouse; level matched at an equidistance of 480 µm) were then compressed and stored in.tiff format. These images were then imported into Fiji Image-J software and amyloid burden was quantified and recorded for the group comparisons. To measure amyloid burden, the sections were converted to 8-bit images followed by a selection of cerebral cortex with a hand-tool outline. Threshold number was applied to highlight all amyloid plaques after running some preliminary analysis with these set of images. Images were then processed through fill holes and watershed plugins. Analyze particles was then applied to collect the amyloid burden and amyloid plaque size for each section, from which collated numbers were collected and compared between groups using Graph Pad Prism software.

### ABX-extraction using solid phase extraction cartridge

Brain homogenization and extracts were performed according to the manufacturer’s protocol (Cat# 8B-S100-UBJ, Phenomenex). In brief, brains were homogenized in Milli-Q water using a tissue homogenizer. The positive control samples (n = 6) were spiked with an individual ABX or ABX-cocktail. After homogenization, the vial was centrifuged at 10000 rpm for 10 min and the supernatant was used for solid phase extraction. The solid phase extraction cartridge was activated using 3 mL methanol followed by 3 mL 0.1% acetic acid and the samples were loaded on the cartridge and washed with 3 mL of 0.1% acetic acid. Analytes were then diluted with methanol (MeOH) followed by evaporation to dryness. The residual samples were then analyzed by LC-MS.

### UPLC-MS of ABX in the brain

Accurate mass data was acquired using ultra high-pressure liquid chromatography-electrospray ionization-quadrupole time-of-flight (+UPLC-ESI-LC-QTOF, Agilent 6540) instrumentation. Samples (5 µL) were separated by C18 (ZORBAX Eclipse Plus C18, 2.1 × 100 mm, 1.8 micron, Agilent) with the following gradient: 0–1 min 0% B (A, 95% H_2_O, 5% MeOH, 0.1% formic acid; B, 100% MeOH), 1-10 min 0-95% B, 10-12 95% B. Data was acquired in centroid mode with source/fragmentor voltage of 120 V, positive mode ion detection between 150–1800 *m/z*, gas temperature of 325 °C, and capillary voltage of 3500 V. The following *m/z* values were extracted: metronidazole, 172.0717 [M + H]^+^; vancomycin, 724.7224 [M + 2H]^+2^; gentamicin, 478.3235 [M + H]^+^; kanamycin, 485.2453 [M + H]^+^; minocycline, 458.1922 [M + H]^+^; colistin, 578.3882 [M + 2H]^+2^; minocycline (internal standard added to all samples), 458.1922 [M + H]^+^. All compounds were detected with <Δ5 ppm compared to calculated exact mass. As previously described, the limit-of-detection (LOD) was calculated for all antibiotics with the following equation: LoD = meanblank +1.645 (SDblank), where the mean blank is estimated from blank replicates containing no antibiotics. The following LODs were determined: metronidazole, 1 µM; vancomycin, 10 µM; gentamicin, 10 µM; kanamycin, 10 µM; minocycline, 10 µM; colistin, 10 µM; minocycline (IS), 1 µM.

### Statistical analysis

GraphPad Prism software was used to run statistical analysis. Shapiro-Wilk normality test was performed for all data sets. All groups in Cecum weight (Fig. 1A), alpha-diversity (Fig. 1C), and Aβ burden (Fig. 2H) datasets passed Shapiro-Wilk normality test and so we used one-way ANOVA to compare multiple groups followed by a Dunnett post-hoc comparison. Only one group out of seven groups, in Faith phylogenetic diversity (ABX cocktail group) and mean Aβ plaque size (kanamycin group) data sets did not pass the normality test. However, as we are interested in comparing the mean values across the groups, we continued parametric analysis ANOVA for these comparisons. A statistical *P* value below 0.05 was considered as a significant difference.

## References

[1] Selkoe DJ (2001). Alzheimer’s disease results from the cerebral accumulation and cytotoxicity of amyloid beta-protein. J. Alzheimers Dis..

[2] Grundke-Iqbal I (1986). Abnormal phosphorylation of the microtubule-associated protein tau (tau) in Alzheimer cytoskeletal pathology. Proc. Natl Acad. Sci. U S A.

[3] Morimoto K (2011). Expression profiles of cytokines in the brains of Alzheimer’s disease (AD) patients compared to the brains of non-demented patients with and without increasing AD pathology. J. Alzheimers Dis..

[4] Patel NS (2005). Inflammatory cytokine levels correlate with amyloid load in transgenic mouse models of Alzheimer’s disease. J. Neuroinflammation.

[5] Wang WY, Tan MS, Yu JT, Tan L (2015). Role of pro-inflammatory cytokines released from microglia in Alzheimer’s disease. Ann. Transl. Med..

[6] Apelt J, Schliebs R (2001). Beta-amyloid-induced glial expression of both pro- and anti-inflammatory cytokines in cerebral cortex of aged transgenic Tg2576 mice with Alzheimer plaque pathology. Brain Res..

[7] McGeer PL, Itagaki S, Tago H, McGeer EG (1987). Reactive microglia in patients with senile dementia of the Alzheimer type are positive for the histocompatibility glycoprotein HLA-DR. Neurosci. Lett..

[8] Zotova E (2011). Microglial alterations in human Alzheimer’s disease following Abeta42 immunization. Neuropathol. Appl. Neurobiol..

[9] Shao Y, Gearing M, Mirra SS (1997). Astrocyte-apolipoprotein E associations in senile plaques in Alzheimer disease and vascular lesions: a regional immunohistochemical study. J. Neuropathol. Exp. Neurol..

[10] Streit WJ, Mrak RE, Griffin WS (2004). Microglia and neuroinflammation: a pathological perspective. J. Neuroinflammation.

[11] Minter MR (2016). Antibiotic-induced perturbations in gut microbial diversity influences neuro-inflammation and amyloidosis in a murine model of Alzheimer’s disease. Sci. Rep..

[12] Dodiya HB (2019). Sex-specific effects of microbiome perturbations on cerebral Abeta amyloidosis and microglia phenotypes. J. Exp. Med..

[13] Minter MR (2017). Antibiotic-induced perturbations in microbial diversity during post-natal development alters amyloid pathology in an aged APPSWE/PS1DeltaE9 murine model of Alzheimer’s disease. Sci. Rep..

[14] Nho K (2019). Altered bile acid profile in mild cognitive impairment and Alzheimer’s disease: Relationship to neuroimaging and CSF biomarkers. Alzheimers Dement..

[15] Soscia SJ (2010). The Alzheimer’s disease-associated amyloid beta-protein is an antimicrobial peptide. PLoS One.

[16] Harach T (2017). Reduction of Abeta amyloid pathology in APPPS1 transgenic mice in the absence of gut microbiota. Sci. Rep..

[17] Sun J (2019). Fecal microbiota transplantation alleviated Alzheimer’s disease-like pathogenesis in APP/PS1 transgenic mice. Transl. Psychiatry.

[18] Kim MS (2020). Transfer of a healthy microbiota reduces amyloid and tau pathology in an Alzheimer’s disease animal model. Gut.

[19] Bonfili L (2018). SLAB51 Probiotic Formulation Activates SIRT1 Pathway Promoting Antioxidant and Neuroprotective Effects in an AD Mouse Model. Mol. Neurobiol..

[20] Kobayashi Y (2017). Therapeutic potential of Bifidobacterium breve strain A1 for preventing cognitive impairment in Alzheimer’s disease. Sci. Rep..

[21] Wang X (2019). Sodium oligomannate therapeutically remodels gut microbiota and suppresses gut bacterial amino acids-shaped neuroinflammation to inhibit Alzheimer’s disease progression. Cell Res..

[22] Bashir ME, Louie S, Shi HN, Nagler-Anderson C (2004). Toll-like receptor 4 signaling by intestinal microbes influences susceptibility to food allergy. J. Immunol..

[23] Hansen CH (2012). Early life treatment with vancomycin propagates Akkermansia muciniphila and reduces diabetes incidence in the NOD mouse. Diabetologia.

[24] Cox LM (2014). Altering the intestinal microbiota during a critical developmental window has lasting metabolic consequences. Cell.

[25] Cho I (2012). Antibiotics in early life alter the murine colonic microbiome and adiposity. Nature.

[26] Desbonnet L (2015). Gut microbiota depletion from early adolescence in mice: Implications for brain and behaviour. Brain Behav. Immun..

[27] Han D (2015). Microbiota-Independent Ameliorative Effects of Antibiotics on Spontaneous Th2-Associated Pathology of the Small Intestine. PLoS One.

[28] Morgun A (2015). Uncovering effects of antibiotics on the host and microbiota using transkingdom gene networks. Gut.

[29] Vogt NM (2017). Gut microbiome alterations in Alzheimer’s disease. Sci. Rep..

[30] Cattaneo A (2017). Association of brain amyloidosis with pro-inflammatory gut bacterial taxa and peripheral inflammation markers in cognitively impaired elderly. Neurobiol. Aging.

[31] Shen L, Liu L, Ji HF (2017). Alzheimer’s Disease Histological and Behavioral Manifestations in Transgenic Mice Correlate with Specific Gut Microbiome State. J. Alzheimers Dis..

[32] Bauerl C, Collado MC, Diaz Cuevas A, Vina J, Perez Martinez G (2018). Shifts in gut microbiota composition in an APP/PSS1 transgenic mouse model of Alzheimer’s disease during lifespan. Lett. Appl. Microbiol..

[33] Brandscheid C (2017). Altered Gut Microbiome Composition and Tryptic Activity of the 5xFAD Alzheimer’s Mouse Model. J. Alzheimers Dis..

[34] Familian A, Boshuizen RS, Eikelenboom P, Veerhuis R (2006). Inhibitory effect of minocycline on amyloid beta fibril formation and human microglial activation. Glia.

[35] Seabrook TJ, Jiang L, Maier M, Lemere CA (2006). Minocycline affects microglia activation, Abeta deposition, and behavior in APP-tg mice. Glia.

[36] Noble W (2009). Minocycline reduces the development of abnormal tau species in models of Alzheimer’s disease. FASEB J..

[37] Biscaro B, Lindvall O, Tesco G, Ekdahl CT, Nitsch RM (2012). Inhibition of microglial activation protects hippocampal neurogenesis and improves cognitive deficits in a transgenic mouse model for Alzheimer’s disease. Neurodegener. Dis..

[38] Sun L (2019). Antibiotic-Induced Disruption of Gut Microbiota Alters Local Metabolomes and Immune Responses. Front. Cell Infect. Microbiol..

[39] Radde R (2006). Abeta42-driven cerebral amyloidosis in transgenic mice reveals early and robust pathology. EMBO Rep..

[40] Bolyen E (2019). Reproducible, interactive, scalable and extensible microbiome data science using QIIME 2. Nat. Biotechnol..

[41] Callahan BJ (2016). DADA2: High-resolution sample inference from Illumina amplicon data. Nat. Methods.

[42] Katoh K, Standley DM (2013). MAFFT multiple sequence alignment software version 7: improvements in performance and usability. Mol. Biol. Evol..

[43] Price MN, Dehal PS, Arkin AP (2010). FastTree 2–approximately maximum-likelihood trees for large alignments. PLoS One.

[44] Weiss S (2017). Normalization and microbial differential abundance strategies depend upon data characteristics. Microbiome.

[45] Faith DP (1992). Conservation evaluation and phylogenetic diversity. Biol. Conserv..

[46] Lozupone C, Knight R (2005). UniFrac: a new phylogenetic method for comparing microbial communities. Appl. Env. Microbiol..

[47] Lozupone CA, Hamady M, Kelley ST, Knight R (2007). Quantitative and qualitative beta diversity measures lead to different insights into factors that structure microbial communities. Appl. Env. Microbiol..

[48] Bokulich NA (2018). Optimizing taxonomic classification of marker-gene amplicon sequences with QIIME 2’s q2-feature-classifier plugin. Microbiome.

[49] McDonald D (2012). An improved Greengenes taxonomy with explicit ranks for ecological and evolutionary analyses of bacteria and archaea. ISME J..

[50] Pedregosa, F. *et al*. Machine Learning in Python. *J. Machine Learning Res.* **5** (2012).

[51] Mandal S (2015). Analysis of composition of microbiomes: a novel method for studying microbial composition. Microb. Ecol. Health Dis..

